# CardIAP: calcium transients confocal image analysis tool

**DOI:** 10.3389/fbinf.2023.1137815

**Published:** 2023-07-14

**Authors:** Ana Julia Velez Rueda, Luis Alberto Gonano, Agustín García Smith, Gustavo Parisi, María Silvina Fornasari, Leandro Matías Sommese

**Affiliations:** ^1^ Departamento de Ciencia y Tecnología, CONICET, Universidad Nacional de Quilmes, Bernal, Argentina; ^2^ Centro de Investigaciones Cardiovasculares, Facultad de Medicina, CONICET, Universidad Nacional de La Plata, La Plata, Argentina

**Keywords:** calcium transient, confocal microscopy, impaired calcium handling, discordance analysis, alternans

## Abstract

One of the main topics of cardiovascular research is the study of calcium (Ca2+) handling, as even small changes in Ca2+ concentration can alter cell functionality (Bers, Annu Rev Physiol, 2014, 76, 107–127). Ionic calcium (Ca2+) plays the role of a second messenger in eukaryotic cells, associated with cellular functions such as cell cycle regulation, transport, motility, gene expression, and regulation. The use of fluorometric techniques in isolated cells loaded with Ca2+-sensitive fluorescent probes allows quantitative measurement of dynamic events occurring in living, functioning cells. The Cardiomyocytes Images Analyzer Python (CardIAP) application addresses the need to analyze and retrieve information from confocal microscopy images systematically, accurately, and rapidly. Here we present CardIAP, an open-source tool developed entirely in Python, freely available and useable in an interactive web application. In addition, CardIAP can be used as a standalone Python library and freely installed via PIP, making it easy to integrate into biomedical imaging pipelines. The images that can be generated in the study of the heart have the particularity of requiring both spatial and temporal analysis. CardIAP aims to open the field of cardiomyocytes and intact hearts image processing. The improvement in the extraction of information from the images will allow optimizing the usage of resources and animals. With CardIAP, users can run the analysis to both, the complete image, and portions of it in an easy way, and replicate it on a series of images. This analysis provides users with information on the spatial and temporal changes in calcium releases and characterizes them. The web application also allows users to extract calcium dynamics data in downloadable tables, simplifying the calculation of alternation and discordance indices and their classification. CardIAP aims to provide a tool that could assist biomedical researchers in studying the underlying mechanisms of anomalous calcium release phenomena.

## Introduction

Cardiovascular diseases (CVD) are the leading cause of global death^1^, and in particular, arrhythmias represent a major portion of these deaths (∼15–20%) (Srinivasan and Schilling, 2018). Because abnormal Ca2+ handling is associated with cardiac arrhythmias, understanding calcium (Ca2+) management is one of the main goals of cardiovascular research (Bers, 2014; Landstrom et al., 2017). Ca2+ release in cardiomyocytes occurs as a consequence of the coordinated opening of multiple Ca2+ release channels (Guatimosim et al., 2002) that can exhibit independent properties and lead to different types of arrhythmogenic events (Chen et al., 2012; Hammer et al., 2014; Sommese et al., 2016). Under certain conditions, such as rapid pacing, there is a change in the amplitude of Ca2+ transients from beat to beat in cardiac myocytes, which is referred to as Ca2+ alternans (Eisner et al., 2006; Qu et al., 2013; Edwards and Blatter, 2014). Interestingly, a particular form of Ca2+ alternans, in which different cell areas alternate out of phase, was found both in the atrial (Blatter et al., 2003) and ventricular myocytes (Aistrup et al., 2006; Aistrup et al., 2009). This phenomenon, termed “subcellular discordant alternans”, has not been quantitatively studied in previous work, and its physiological consequences remain unclear. This term was previously used to describe a phenomenon in which different regions of cardiac tissue exhibit an alternating sequence of action potential durations that are not in phase (Sato et al., 2013). Quantification of changes in the Ca2+ cycle brings us closer to understanding this phenomenon and its consequences. In this sense, image-based techniques could provide valuable spatial and temporal information about intracellular Ca2+ management of cardiomyocytes (Cannell et al., 1987).

Image-based techniques are widely used tools in biomedical research because they provide spatial and temporal information in various domains and, in particular, on intracellular Ca2+ management of cardiomyocytes. Among the methods that provide more detailed information about the kinetics of Ca2+ movements in the cell, confocal microscopy is the most commonly used (Price et al., 2014). However, there are only a few open tools available for its analysis (Schneider et al., 2012; JuholaPenttinen et al., 2015; PenPnen et al., 2015; Giovannucci et al., 2019; PsarasMargara et al., 2021; Pascalin et al., 2022), and are usually used for general image analysis or the study of specific phenomena or even for the analysis of calcium handling in different types of cells (Kolar et al., 2021; Pachitariu et al., 1507).

In general, the analysis of image data from confocal microscopy consists of three phases: the extraction of data from the images, the statistical-mathematical processing of these data, and their visualization. These phases currently require the use of more than one software, making large-scale analysis difficult. To name just one possible manual workflow: Manual data collection requires researchers to open the images, preprocess them, and extract numerical values of the average intensity of each image, e.g., using a combination of ImageJ software for visualization and Excel (or similar) for data analysis. Once these data are available and properly organized in a spreadsheet, specialized software for statistical data analysis and data visualization will be needed. Depending on the experience of the researcher, these steps can be performed with a fully automated tool such as Origin or with homegrown code such as Matlab, which may require paid licenses.

Here we introduce CardIAP^2^, a free open-source tool that enables the analysis of a range of Ca2+ handling phenomena using a single or multiple confocal microscopy images. Its distribution on the Internet as a web app allows the user to easily manipulate the desired images to obtain representative amplitude and kinetics data. Our goal is to provide a user-friendly tool that enables large-scale analysis of confocal microscopy images.

## Materials and methods

### Calcium peaks identification and data processing

**FIGURE 1:**
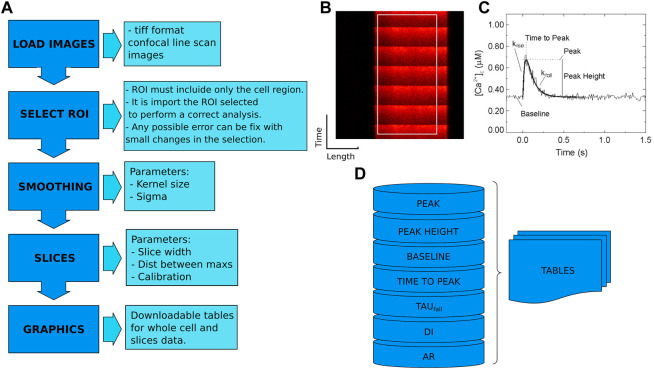
**(A)**. Cardilib workflow: Automatic image processing follows a sequence of packages (image loading, smoothing, selection ROI, separation into slices, analysis, and display) described in the ‘Calcium peak identification and data processing’ section. **(B)**. Reference image of a linescan of isolated cardiomyocyte from rat heart with an example of ROI. **(C)**. Representative calcium transient where the measured parameters are given. From these data, CardIAP calculates time to half amplitude, time to peak and amplitude normalized to baseline, unconformity indices (DI), and alternans ratios (AR). **(D)**. The analysis is based on the detection of the peaks. These data are organized into tables for the entire cell and each slice performed.

The CardIAP web application uses Cardilib for image analysis. This is a Python library developed by the authors of this paper for biomedical image analysis that can be installed and used via pip^3^ on all operating systems (https://pypi.org/project/cardilib/). To analyze each image, CardIAP converts it into a three-dimensional matrix corresponding to the size of the image in pixels and the intensity of each pixel (Figure 1). From this matrix, the image is cropped in the region selected by the user with the cropper displayer (region of interest, ROI), and CardIAP analyzes the calcium transients by locating the position of the local intensity maximum.

The algorithm finds the peaks with the help of the first order difference (Negri and Vestri, 2017). These potential peaks are filtered based on the minimum distance between them, expressed in pixels, and an intensity threshold based on the average of the data set. The user has the option of retrieving the intensity average of the entire selection over the time series, or portioning it and applying the analysis to each of the slices. CardIAP also provides the user with the relative amplitude of each calcium transient, calculated from the difference between the maximum and minimum, divided by the minimum value. To calculate the decay rate (tau value) of each peak, the time series data are truncated from the maximum to the next minimum and applied to the least squares solution of the linear matrix.

Local and global alternans ratios are calculated by taking the absolute value of the difference between the maximum values of the successive time series and normalizing by the maximum value between the two peaks:

$$\left[AR=abs\left(Peak1-Peak2\right))/\max\left(Peak1,Peak2\right)\right]$$

By using + dF/dt max during the upstroke of the Ca2+ transient in the calculation of local alternans ratios, it is possible to measure the extent of local Ca2+ release before the change in position of the local Ca2+ signal promoted by cell contraction. The discordance index is the standard deviation of the local alternans ratios (Sommese et al., 2022) (Supplementary Figure S1).

### CardIAP web distribution implementation

CardIAP is also distributed as a web application implemented with the Voilá framework^4^, which enables the creation of standalone web applications and dashboards from Jupyter notebooks. The CardIAP web application takes user-supplied images as input and allows the images to be cropped interactively^5^ before analysis. The application reads the image format TIFF, JPG or even PNG from disk. Users can also save the results as CSV tables with the data from the full-cell and slices analysis. In addition, a public docker image of CardiAP is also available in DockerHub^6^. The tutorial and documentation for the app can be found at https://cardiap.github.io/, a GitHub page of the original code repository.

### CardIAP access and user interface

The Upload button on the CardIAP user interface (Supplementary Figure S2A) allows users to upload one or more images from their local hard drives. After uploading the images, users can crop each image using the image display and the width and height selectors (Supplementary Figure S2B). Once the crop sizes are saved, CardIAP prints them to the screen and displays the options for smoothing and analysis settings: Kernel Size, Sigma, Slice Width, Peaks dist (distance in pixels between peaks), and Calibration. In short, CardIAP allows the user to apply a bilateral filter to smooth the image. The kernel size is the diameter of each pixel neighborhood used in the filtering. A value of zero allows the image not to be filtered. A filter sigma in coordinate space and color space is a measure of the geometric proximity and photometric similarity of the images. A larger value of the parameter means that pixels further away will affect each other as long as their colors are close enough. A value of zero in this parameter allows the image not to be filtered.

The slice width value, which takes values greater than zero (pixels), allows the user to slice the region of interest (ROI) for calcium local transient analysis into pieces of the specified size in pixels. This is particularly important because Ca2+ release results from the opening of ryanodine receptor channels (RyR2), which are clustered in regions of 2 μm and can fire independently, resulting in dysynchronous or discordant patterns (Qu et al., 2013). Also, the value for the distance between peaks (Peaks dist.) helps CardIAP to improve the threshold for the maximum intensity positions. This parameter takes positive numerical values, and its value depends on the acquisition rate and stimulus frequency. In our use cases, we use 200 for a frequency of 1 Hz, 70 for 3 Hz, 50 for 4 Hz, and 40 for 5 Hz. And finally, it is possible to calibrate the value for converting the position of the pixels into time data.

The results for each analyzed image are displayed in different tabs (Supplementary Figure S3). The user can retrieve the information for all slices and the entire image analysis separately (Supplementary Figure S3). CardIAP provides amplitude and intensity plots as well as downloadable tables with all analysis results. The data of the individual slices can be visualized on the “Slices detail” tab.

### Calcium imaging dataset building

The images used for the development and testing of CardIAP were acquired with a confocal microscope. For this purpose, cardiomyocytes loaded with Fluo-4 were imaged with a Zeiss 410 inverted confocal microscope (LSMTech, Pennsylvania, United States of America) equipped with a 63x, 1.4 NA oil immersion objective. Ventricular cardiomyocytes were isolated from hearts of male Wistar rats according to the protocol described by Louch et al. in Methods in cardiomyocyte isolation, culture, and gene transfer (Negri and Vestri, 2017). Fluo-4 is a Ca2+ indicator designed for use with visible light sources in flow cytometry and confocal laser scanning microscopy (Szlovák et al., 2021). Fluorescence excitation was performed with a 488 nm argon laser, and light emitted at 500–550 nm was collected. Fluo-4 fluorescence was recorded in line scan mode, with a line of 512 pixels placed along the long axis of the cell. In addition, each acquisition consisted of 512 consecutive line scans (4.3 ms per line) stacked to create space-time profiles. All experiments were performed at room temperature (24°C).

The experiments performed followed the Guide for the Care and Use of Laboratory Animals (NIH Publication No.85-23, revised 2011) and were approved by the Institutional Animal Care and Use Committee of La Plata University School of Medicine.

### Statistical analysis

To evaluate the performance of our application, we analyzed confocal microscopy images (n = 50) manually and with CardIAP. Continuous variables were expressed as mean ± standard deviation and evaluated with a simple Student’s t-test. The Kolmogorov-Smirnov test was used to compare nonparametric distributions. Spearman’s correlation coefficients were used to measure the relationship between two variables. And Levene’s test was used to test the homogeneity of variance of samples. A *p*-value <of 0.05 was considered significant.

## Results

**FIGURE 2:**
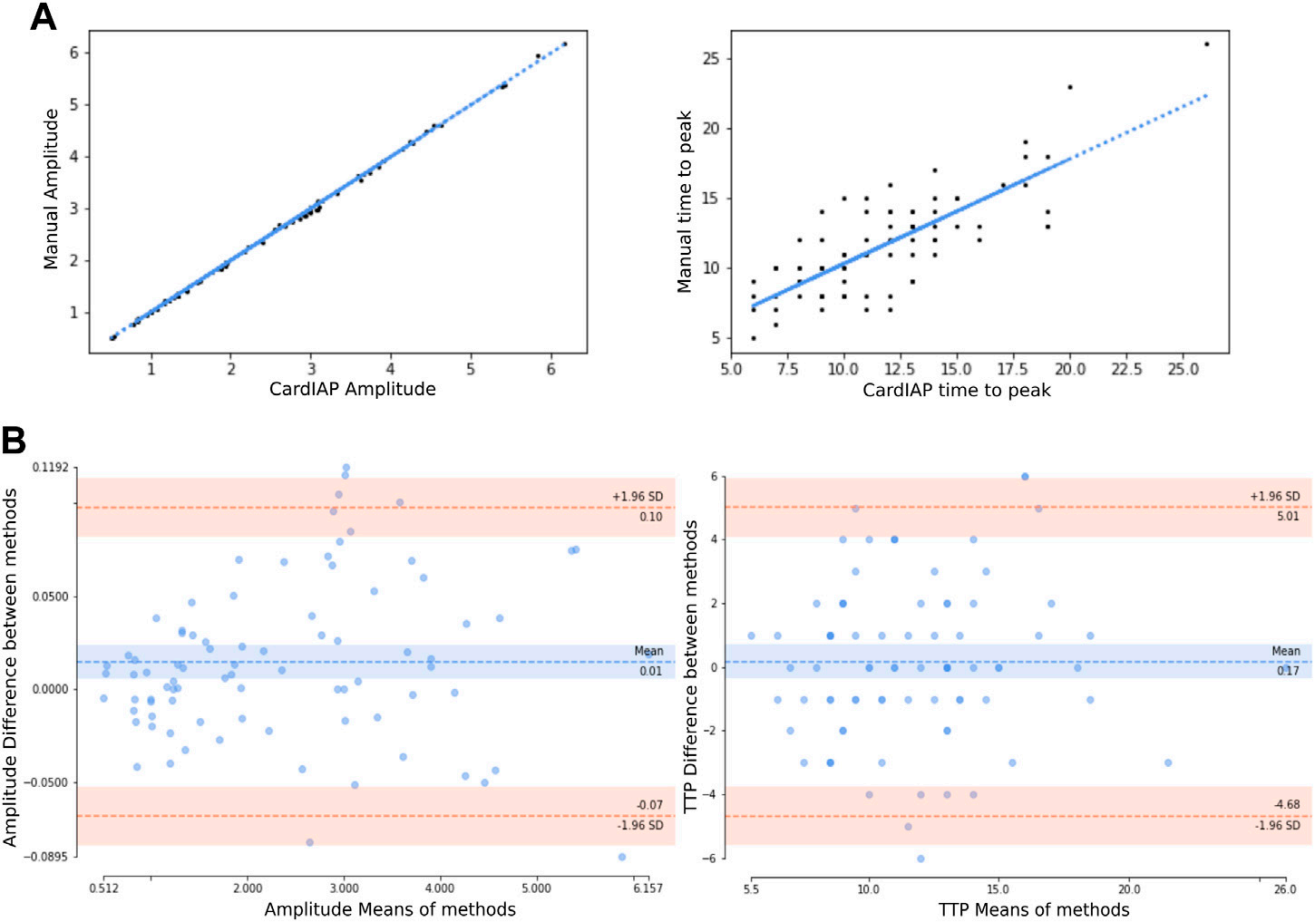
**(A)**. At left, correlation plot of the amplitude of calcium transient manually measured and using CardIAP. At right, correlation plot of time to peak of calcium transient manually measured and using CardIAP. **(B)**. At left, Bland-Altman plot of the amplitude of calcium transient manually measured and using CardIAP. At right, Bland-Altman plot of time to peak calcium transient, measured manually and using CardIAP. The *Y*-axis represents the difference between two paired measurements. The blue line is the mean difference. The red line is the ±1.96 standard deviations from the mean difference. The *X*-axis represents the mean of the two measurements.

To better understand the performance of our software, we performed a comparison between the values calculated with CardIAP and the transient amplitude and time-to-peak (TTP) data manually extracted from the same images with an image analysis program (ImageJ). Figure 2A shows the correlations between the two methods. Panel A shows the linear regression correlating the amplitude values obtained with CardIAP and manually. The Spearman coefficient is 0.99 (*p*-value <0.0001). It also shows the correlation of normalized TTP values, which has a Spearman coefficient of 0.71 (*p*-value <0.0001). From these results, we can infer that both methods provide related data that differ greatly.

To find out whether the pixel intensity and time measurements made with CardIAP have a bias or not, we determined the paired difference between the values calculated with both methods. For the amplitude measurement, the mean value of the difference between the methods is 0.014 ± 0.042 (Figure 2B) and for the TTP it is 0.166 ± 2.487 (Figure 2B). This shows that there is no significant bias in the TTP measurement (*p*-value >0.05), but a small bias in the amplitude measurement (*p*-value <0.05). While this observation is not biologically relevant, it can be explained by the noisy signal measurement causing the trend of a slightly higher measurement with CardIAP compared to manual measurement.

The two measured parameters do not differ significantly in variance when comparing the manual and CardIAP measurements (Levene’s test *p*-value >0.05). For amplitude, the variances are 1.80 for CardIAP and 1.79 for the manual measurement; for TTP, the variances are 14.38 for CardIAP and 13.08 for the manual measurement. Thus, we can conclude that the dispersion around the mean values is the same for both methods, i.e., not only do they have similar central values, but they also fluctuate around them in the same way.

### Application case

Repolarization alternans, especially when it becomes spatially discordant, is a harbinger of sudden cardiac death (Walker and Rosenbaum, 2003). As mentioned previously, alternans is a phenomenon in which the amplitude of Ca2+ transients changes from beat to beat in cardiac myocytes. Discordant alternans is attributed to phase-shifted alternation of local Ca2+ release in the cell. Several mechanisms have been proposed linking spatially discordant alternans formed by fast pacing to altered intracellular Ca2+ cycling, and in which Ca2+ transients and action potential duration alternans are electromechanically concordant (Sato et al., 2013). Fluctuations of the Ca2+ cycle may determine the Ca2+ alternans phase because the amplitude of Ca2+ alternans is low in the early phases of stimulation. Therefore, different regions of a cardiac myocyte typically develop Ca2+ alternans that oppose each other during the early phases of stimulation. These subcellular patterns then gradually coarsen due to interactions with membrane voltage, forming stationary, spatially discordant voltage and Ca2+ alternans at the tissue scale.

Precise measurement of indicators and indices of the phenomena associated with altered calcium cycling will allow us to deepen the study of the mechanisms underlying arrhythmias.

**FIGURE 3:**
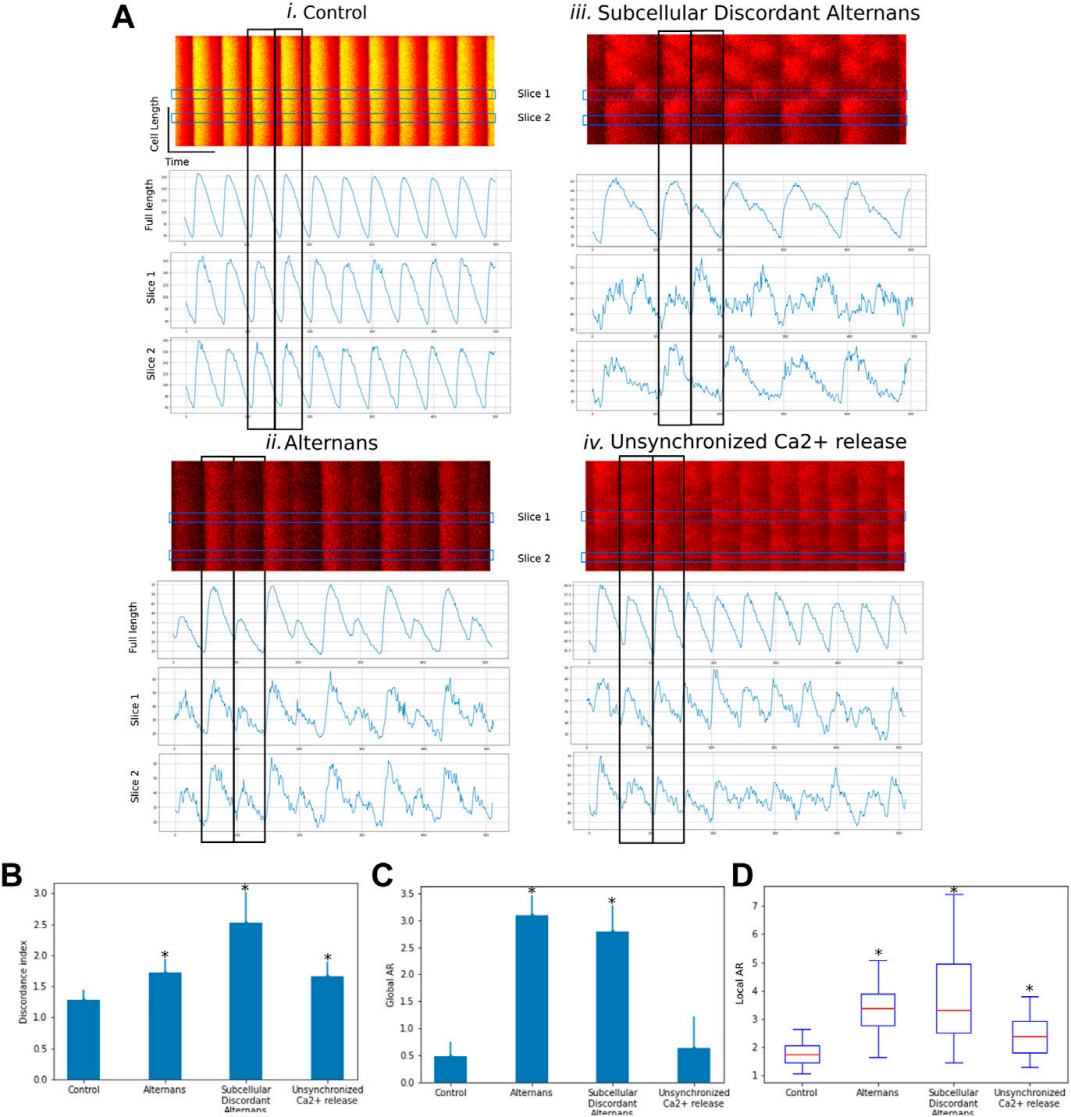
**(A)**. Representative confocal line scan images of four different cases (control, alternans, subcellular discordant alternans, unsynchronized Ca2+ release). Line plots show intensity variation of fluorescence along time for full length and two sections. **(B)** Bar graph of Discordance Index (DI) for each case. **(C)** Bar graph of global alternans ratio (AR) for each case. **(D)** Boxplot of local alternans ratio (AR) for each case. Data are means ± SD. *p < 0.05 compared with control.

Using CardIAP, we compared the discordance indexes (DI) and alternans ratios (AR) of confocal images of cardiomyocytes showing different behaviors associated with abnormal calcium cycling (concordant alternans, discordant alternans). We analyzed 40 confocal images isolated from 10 rat hearts previously characterized in Sommese et al. (2022) and classified as control, alternans, and discordant alternans (Sommese et al., 2022). These results show that the CardIAP measurement of DI and AR can distinguish between different phenomena. Figure 3A shows confocal line images of representative rat cardiomyocytes under four conditions: Control, Alternans, Alternans discordant, and unsynchronized Ca2+ release. Fluorescence intensities as a function of time are plotted below each image. Each peak represents calcium efflux and reuptake. These data were obtained by electrical stimulation of isolated cardiomyocytes. To show the alternating behavior, the peaks are plotted as intensity measures for the entire length of the cell and for portions along the length of the cell. In case i, it can be seen that the peaks of the whole cell coincide in time with those of the individual sections and have similar intensity values between one peak and the subsequent one. In case ii, all peaks coincide, but the amplitude of two successive peaks is alternating as their amplitude alternates between high and low amplitude; this behavior can be observed in all sections as in the cells of the whole length. Case iii also shows that the peaks of the entire cell alternate between high and low amplitudes. This effect does not coincide in time along the entire length of the cell, so that the high amplitude peaks do not coincide with the low amplitude peaks for each slice. In case iv, we can observe something similar to case iii, but in a less ordered and pronounced way, where the amplitudes of the peaks alternate and there are differences between different parts.

When we compare the DI of the different phenomena, we observe a higher DI in the images showing changes in the synchronism of Ca2+ release, which is even higher in the example of subcellular discordant alternans (Figure 3B). In contrast, when we compare the global AR for each case, we can see that the examples manifesting the alternans phenomenon, either concordant or discordant, have significantly higher values (Figure 3C). In the analysis of case iv, the alternans phenomenon is not consistently observed throughout the image or over time, as reflected in the global AR values, which are similar to those of the control case. But when evaluating the local AR, i.e., in 2 μm sections, the resolution of the analysis allows highlighting the unsynchronized Ca2+ release, which has higher values than in the control case, but not as much as in the examples with clear alternans (Figure 3D). This is accompanied by the larger DI seen in Figure 3B.

These results demonstrate that CardIAP is capable of measuring Ca2+ transients with sufficient precision to assess and discriminate changes in Ca2+ cycling by microscopy assays.

## Conclusion

Here we present CardIAP, a free tool to facilitate the analysis of multiple line scan images acquired using calcium transient microscopy techniques. It allows easy conversion of cell images into time-series plots to accurately detect transient peaks. CardIAP measures the intensity and kinetic parameters of the peaks quickly and transparently, giving the user control over the processes that run automatically.

Our tool offers the user the possibility to analyze a number of images in parallel, but to individually select the area of interest. Unlike manual image processing, this option allows the study to be repeated and systematized, avoiding bias when reading the graphs. CardIAP thus saves the user from having to use multiple tools, reducing analysis time and the resources to be used. Moreover, as explained above, CardIAP offers the user the possibility to split each image into parts and repeat the extraction of the characteristic parameters.

Easy retrieval of calcium dynamics data by CardIAP facilitates not only the study of calcium transients, but also other calcium release phenomena (Edwards and Blatter, 2014; Skardal et al., 2014; Hohendanner et al., 2016), such as calcium waves and sparks, or to study anomalous calcium release phenomena such as alternation, discordance, and dyssynchrony. CardIAP also provides local and global alternance ratios and discordance indices for each pair of calcium transients.

Furthermore, as we have demonstrated using biologically relevant cases, our application provides a novel way to detect and evaluate concordant and discordant changes relevant to cardiomyocyte Ca2+ cycling studies. Because these indices are not available in any other image analysis software and manual processing requires more time and resources, CardiAp promises to be an invaluable tool for researchers in this field (Louch et al., 2011).

## Data Availability

The raw data supporting the conclusion of this article will be made available by the authors, without undue reservation.

## Footnotes

1 CardiAP project: https://github.com/CardiAP/CardiAp

2 cardilib: https://pypi.org/project/cardilib/

3
https://www.healthdata.org/gbd/2019

4 Voilà: https://voila.readthedocs.io/en/stable/index.html

5 Interactive crop: https://pypi.org/project/interactivecrop/

6 CardiAP public docker images: https://hub.docker.com/r/ajvelezrueda/cardiap
