# Supplementary material for: CardIAP: calcium transients confocal image analysis tool
Source: Front Bioinform. 2023 Jul 14;3:1137815. doi: 10.3389/fbinf.2023.1137815 (PMC10381969; doi:10.3389/fbinf.2023.1137815)
Supplement: Supplementary file 1 [file DataSheet1.PDF]

Supplementary Material

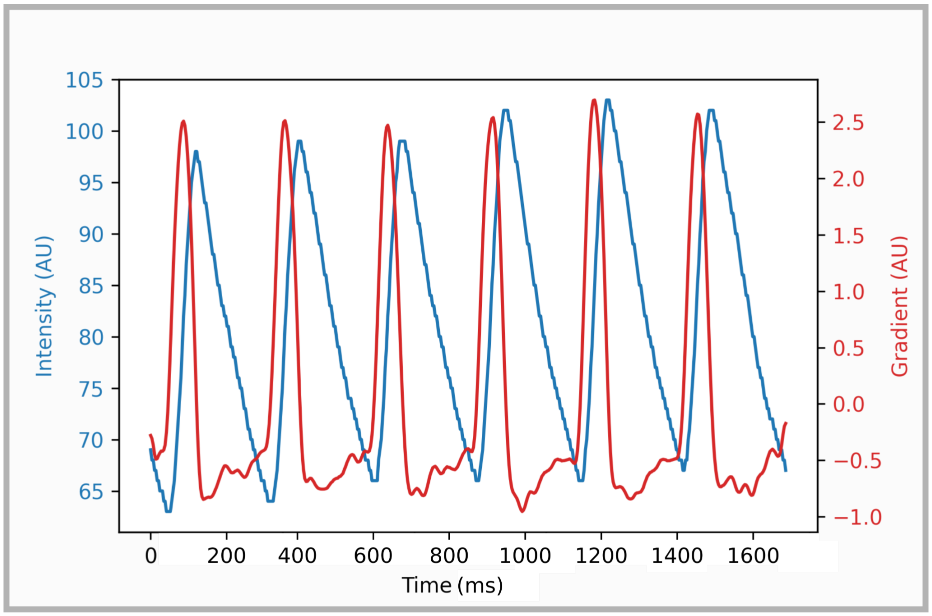

Supplementary Figure 1. Intensity data gradient. The maximum values of the gradient (red line) accompany the data of the corresponding calcium transients (blue line) and precede the contraction.

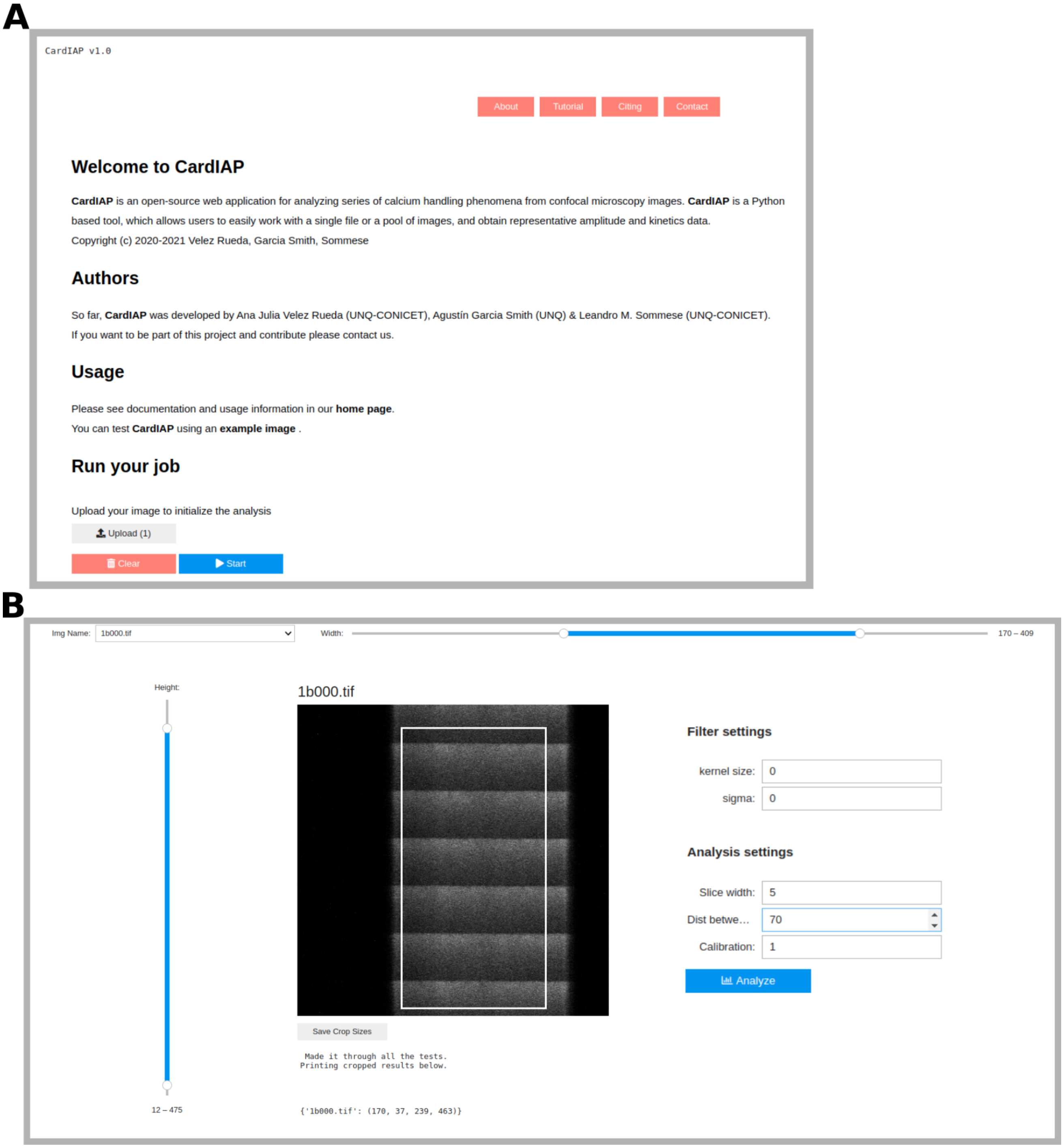

Supplementary Figure 2. **A.** CardIAP home page. **B.** Once the user starts uploading the image, Panel B is displayed to allow cropping of the image. After saving the size of ROI, you can set the smoothing and analysis parameters.

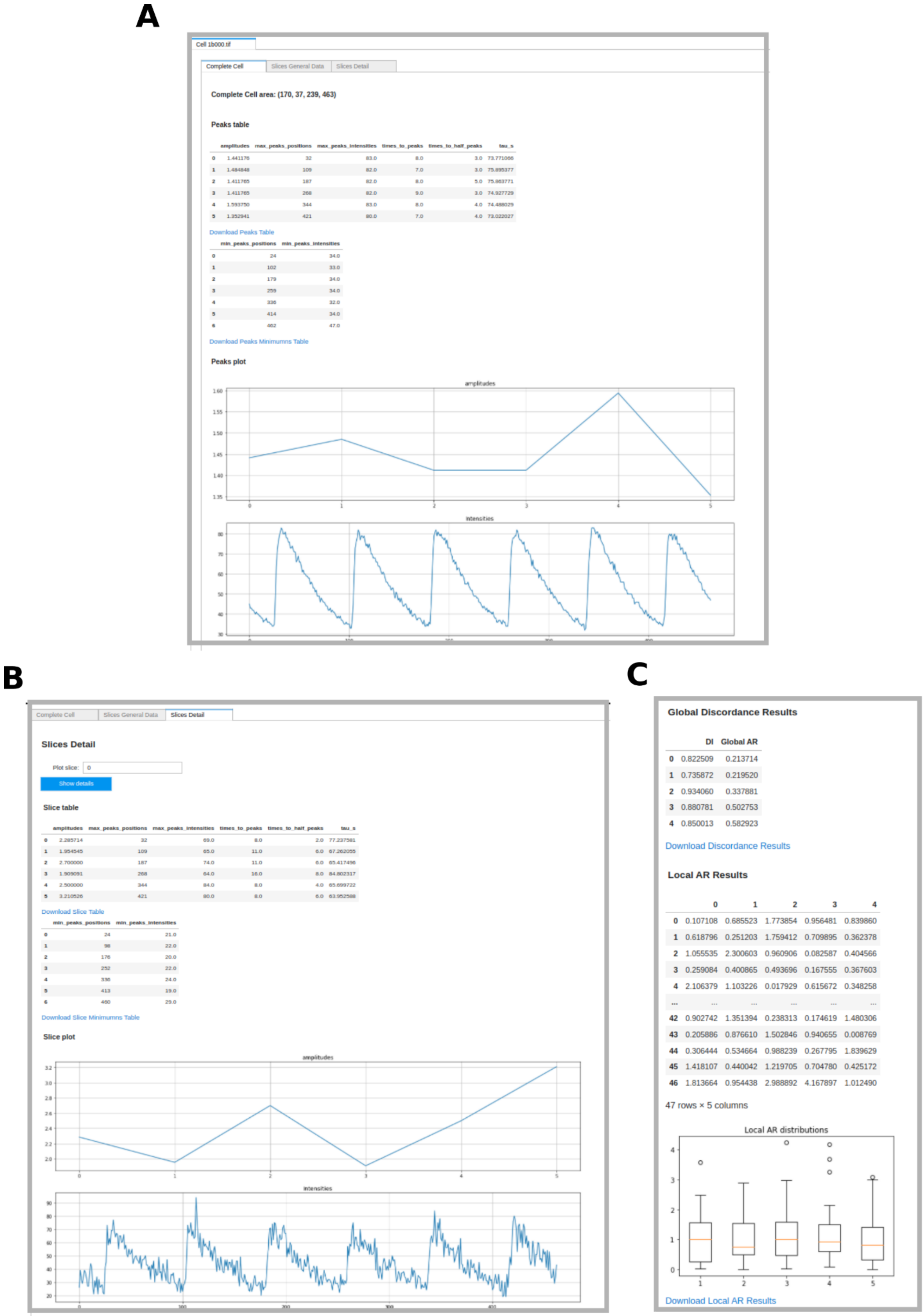
A
Cell 1b000.tif
Complete Cell
Slices General Data
Slices Detail
Complete Cell area: (170, 37, 239, 463)
Peaks table
Download Peaks Table
Download Peaks Minimumns Table
Peaks plot
amplitudes
intensities
B
Slices Detail
Plot slice:
Show details
Slice table
Download Slice Table
Download Slice Minimumns Table
Slice plot
C
Global Discordance Results
DI Global AR
0 0.822509 0.213714
1 0.735872 0.219520
2 0.934060 0.337881
3 0.880781 0.502753
4 0.850013 0.582923
Download Discordance Results
Local AR Results
0 1 2 3 4
0 0.107108 0.685523 1.773854 0.956481 0.839860
1 0.618796 0.251203 1.759412 0.709895 0.362378
2 1.055535 2.300603 0.960906 0.082587 0.404566
3 0.259084 0.400865 0.493696 0.167555 0.367603
4 2.106379 1.103226 0.017929 0.615672 0.348258
... ...
42 0.902742 1.351394 0.238313 0.174619 1.480306
43 0.205886 0.876610 1.502846 0.940655 0.008769
44 0.306444 0.534664 0.988239 0.267795 1.839629
45 1.418107 0.440042 1.219705 0.704780 0.425172
46 1.813664 0.954438 2.988892 4.167897 1.012490
47 rows × 5 columns
Local AR distributions
Download Local AR Results

Supplementary Figure 3. **A.** This panel shows the first rows of the results of the complete cell analysis. Below each table is a link to download the results. A graph of average intensity and peak amplitude is displayed above the image to help the user visualize the intensity data. **B.** If you click on the parent label, you can view the results of the different images and the sections of each image.**C.** Downloadable table of global and local alternans ratios and discordance indices. The distribution of the local alternans ratio is presented in boxplots.
